# CcpA-Independent Glucose Regulation of Lactate Dehydrogenase 1 in *Staphylococcus aureus*

**DOI:** 10.1371/journal.pone.0054293

**Published:** 2013-01-14

**Authors:** Adrianne K. Crooke, James R. Fuller, Markus W. Obrist, Sarah E. Tomkovich, Nicholas P. Vitko, Anthony R. Richardson

**Affiliations:** Department of Microbiology and Immunology, University of North Carolina at Chapel Hill, Chapel Hill, North Carolina, United States of America; Institut Pasteur, France

## Abstract

Lactate Dehydrogenase 1 (Ldh1) is a key enzyme involved in *Staphylococcus aureus* NO·-resistance. Full *ldh*1-induction requires the presence of glucose, and mutants lacking the Carbon-Catabolite Protein (CcpA) exhibit decreased *ldh*1 transcription and diminished Ldh1 activity. The redox-regulator Rex represses *ldh*1 directly by binding to Rex-sites within the *ldh*1 promoter (P*_ldh_*_1_). In the absence of Rex, neither glucose nor CcpA affect *ldh*1 expression implying that glucose/CcpA-mediated activation requires Rex activity. Rex-mediated repression of *ldh*1 depends on cellular redox status and is maximal when NADH levels are low. However, compared to WT cells, the Δ*ccpA* mutant exhibited impaired redox balance with relatively high NADH levels, yet *ldh*1 was still poorly expressed. Furthermore, CcpA did not drastically alter Rex transcript levels, nor did glucose or CcpA affect the expression of other Rex-regulated genes indicating that the glucose/CcpA effect is specific for P*_ldh_*_1_. A putative catabolite response element (CRE) is located ∼30 bp upstream of the promoter-distal Rex-binding site in P*_ldh_*_1_. However, CcpA had no affinity for P*_ldh_*_1_ *in vitro* and a genomic mutation of CRE upstream of P*_ldh_*_1_ in *S. aureus* had no affect on Ldh1 expression *in vivo.* In contrast to WT, Δ*ccpA S. aureus* preferentially consumes non-glycolytic carbon sources. However when grown in defined medium with glucose as the primary carbon source, Δ*ccpA* mutants express high levels of Ldh1 compared to growth in media devoid of glucose. Thus, the actual consumption of glucose stimulates Ldh1 expression rather than direct CcpA interaction at P*_ldh_*_1_.

## Introduction

*Staphylococcus aureus* is an important human pathogen causing disease ranging from mild skin and soft tissue infections to severe invasive sepsis, pneumonia, osteomyelitis and endocarditis [1]. The prevalence of multi drug-resistant strains, particularly those resistant to Methicillin (MRSA), complicates effective treatment in many cases [2]. While drug-resistance limits treatment options, the high virulence potential of *S. aureus* renders even immunocompetent hosts exceedingly susceptible to this pathogen. This is, in part, due to the ability of *S. aureus* to resist nearly every facet of host immunity. Indeed, *S. aureus* encodes many factors that limit the efficacy of opsonophagocytosis, cationic peptides, complement and reactive oxygen species [3]. Consistent with this, *S. aureus* is also remarkably resistant to nitric oxide (NO·), a lipophilic radical that serves as one of the most broad-spectrum antimicrobial effectors of the innate immune system [4], [5]. NO·-resistance distinguishes *S. aureus* from most other bacterial species, including closely related but less pathogenic members of the Staphylococci [6].

*S. aureus* NO·-resistance hinges upon the ability of this organism to metabolically adapt to the cytotoxic effects of host NO· [6]. NO· attacks many metabolic enzymes by targeting active sites composed of iron-sulfur clusters, redox-active thiols and heme motifs [7], [8]. Consequently, NO· is a potent antagonist of aerobic respiration in the host as well as in invading microbes [6], [9]. In response to NO·, *S. aureus* shifts into a metabolic state heavily reliant on fermentative metabolism to combat the negative effects of NO· on respiration [4], [5]. A key enzyme in this response is a lactate dehydrogenase encoded by *ldh*1, a *S. aureus*-specific allele not found in other species of Staphylococci [6]. Ldh1 catalyzes the reduction of pyruvate to L-lactate with the concomitant oxidation of NADH to NAD^+^ for the purpose of restoring redox balance, an essential reaction for cells unable to respire. Consistent with its role in redox-balance, *ldh*1 is normally repressed by the redox-sensing regulator Rex [10]. Rex binds to the *ldh*1 promoter (P*_ldh_*_1_) and limits its expression until the level of NADH rises. The binding of NADH to Rex diminishes DNA binding affinity, effectively leading to the derepression of *ldh*1 as well as many other redox-balancing fermentative enzymes [10], [11]. However, during both NO·-stress and anaerobic fermentation, Ldh1-catalyzed L-lactate production serves as the primary source of redox balance for *S. aureus* [6].

It has also been suggested that Ldh1 expression requires the presence of glucose in addition to redox imbalance [12]. Glucose-mediated induction of Ldh activity in bacteria is not uncommon. For instance in many bacteria, including members of Lactococci and Streptococci, glucose consumption results in high intracellular levels of fructose-1,6-bisphosphate (FBP) an allosteric activator of Ldh that is essential for activity [13]. The binding of FBP stabilizes tetramer formation and improves substrate binding. Many other forms of bacterial Ldh enzymes act independently of FBP and it has been suggested that these enzymes possess intrasubunit salt bridges that obviate the need for FBP allosteric interations [14]. *S. aureus* Ldh1 is such an enzyme and consequently does not require FBP for activity [6]. However, in Gram-positive bacteria, high FBP levels resulting from glucose consumption can lead to changes in gene expression in addition to modulating enzyme activity. FBP accumulation drives additional phosphorylation of Histidine-containing phosphocarrier protein (Hpr) on a conserved serine residue (Ser_46_) through activation of the kinase activity of Hpr-kinase (HprK) [15]. The resulting P∼Ser_46_-HPr can then directly interact with the staphylococcal catabolite control protein A, CcpA [16]. CcpA-P∼Ser_46_-HPr then activates the expression of glycolytic genes and represses TCA cycle and gluconeogenic gene expression allowing for maximal use of available glycolytic carbon sources [17]. In addition, CcpA mediates the effects of glucose on the expression of some *S. aureus* virulence factors including Protein A and FnbB [17]. Here we test whether high intracellular FBP levels activate *ldh*1 transcription by signaling catabolite control through CcpA in *S. aureus*, rather than through allosteric activation of Ldh1. Indeed, a putative catabolite response element (CRE) site, the high-affinity CcpA-P∼Ser_46_-HPr binding site, can be identified upstream of *ldh*1. However, our results show that utilization of glucose stimulates *ldh*1 expression but that this induction is not directly mediated by CcpA.

## Materials and Methods

### Bacterial Strains and Culture Conditions

Mutant strains used in this study are listed in Table 1. *S. aureus* was cultivated in Brain Heart Infusion medium or in chemically defined medium (PN medium) in which primary carbon sources could be modified. Briefly, PN is a phosphate-buffered medium composed of a primary carbon source, nucleobases (Adenine, 5 mg/L; Guanine, 5 mg/L; Cytosine, 5 mg/L; Uracil 5 mg/L and Thymine 20 mg/L), free amino acids (Ala, 60 mg/L; Arg, 70 mg/L; Asp, 90 mg/L; Cystine, 20 mg/L; Glu 100 mg/L; Gly, 50 mg/L; His, 30 mg/L; Iso, 30 mg/L; Leu, 90 mg/L; Lys, 70 mg/L; Met, 10 mg/L; Phe, 40 mg/L; Pro, 10 mg/L; Ser, 30 mg/L; Thr, 30 mg/L; Trp, 10 mg/L; Tyr, 50 mg/L; and Val, 80 mg/L), vitamins (thiamine, 1 mg/L; niacin, 1.2 mg/L; biotin, 5 µg/L; and pantothenate, 250 µg/L), FeCl_3_ at 8 mg/L, MgSO_4_ at 2.5 mg/L and trace elements (ZnCl, 70 µg/L; MnCl, 63 µg/L; Boric Acid, 6 µg/L; CoCl_2_, 190 µg/L; CuCl_2_, 2 µg/L; NiCl_2_, 13 µg/L and Na_2_MoO_4_, 31 µg/L). Antibiotic selection in *S. aureus* (*E. coli*) was performed using the following concentrations: ampicillin (100 µg/ml), chloramphenicol 20 µg/ml, kanamycin 50 µg/ml (50 µg/ml), spectinomycin 100 µg/ml (500 µg/ml), erythromycin 5 µg/ml (300 µg/ml). Growth was monitored as change in absorbance (660 nm) assessed using a Tecan infinite M200 plate reader in 200 µl cultures within a 96 well plate (100 µl headspace). Bacterial cultures used for total RNA isolation, NAD^+^/NADH ration determination and Ldh enzyme assays were cultivated in 50 ml of BHI medium shaking (250 rpm) in 250 ml Erlenmeyer flasks to an OD_660_ ∼ 1.0. For lag phase analyses, 5 ml overnight cultures (BHI) of WT and Δ*rex S. aureus* COL were washed twice in PBS, diluted 1∶1000 in fresh defined medium and growth was monitored in 96 well plates (200 µl cultures, 100 µl headspace). Viable cfu in inocula were enumerated to ensure equal numbers of live bacteria were seeded.

**Table 1:** Strains, Plasmids and Primers.

Strain	Description	Reference
RN4220	Methicillin Sensitive Restriction Deficient *S. aureus*	[22]
Newman	Methicillin Sensitive Clinical Isolate	[23]
COL	Methicillin Resistant Clinical Isolate	[24], [25]
AR0168	*S. aureus* COL Δ*ldh*2::Km^R^	[4]
AR0172	*S. aureus* Newman Δ*ldh*2::Km^R^	[4]
AR0212	*E. coli* DH10B+pCN52 (GFP fusion vector)	This Study
AR0352	*S. aureus* COL Δ*rex*::Km^R^	This Study
AR0407	*S. aureus* COL Δ*ccpA*::Sp^R^	This Study
AR0413	*S. aureus* RN4220+ pJF115 (p*_ldh_*_1_::GFP fusion 1)	This Study
AR0414	*S. aureus* RN4220+ pJF116 (p*_ldh_*_1_::GFP fusion 2)	This Study
AR0415	*S. aureus* RN4220+ pJF117 (p*_ldh_*_1_::GFP fusion 3)	This Study
AR0416	*S. aureus* RN4220+ pJF118 (p*_ldh_*_1_::GFP fusion 4)	This Study
AR0438	*S. aureus* COL Δ*rex*::Km^R^ Δ*ccpA*::Sp^R^	This Study
AR0450	*S. aureus* RN4220+ pJF120 (p*_rpoD_*::GFP fusion)	This Study
AR0452	*S. aureus* RN4220+ pJF122 (p*_ldh_*_1_::GFP fusion)	This Study
AR0480	*S. aureus* RN4220 Δ*ccpA*::Sp^R^+pJF120 (p*_ldh_*_1_::GFP fusion)	This Study
AR0482	*S. aureus* RN4220 Δ*ccpA*::Sp^R^+pJF122 (p*_rpoD_*::GFP fusion)	This Study
AR0614	*S. aureus* COL p*_ldh_*_1_-CRE^*^	This Study
AR0616	*S. aureus* COL Δ*ldh*2::Km^R^ p*_ldh_*_1_-CRE^*^	This Study
AR0717	*E. coli* XL-1 Blue harboring pMWO-101 (N’ His_6_-Rex)	This Study
AR0719	*E. coli* XL-1 Blue harboring pMWO-100 (C’ CcpA-His_6_)	This Study
AR08XX	*S. aureus* Newman Δ*ccpA*::Sp^R^ Δ*ldh*2::Km^R^	This Study

Plasmid	Description	Reference
pBT2ts	*E. coli*/*S. aureus* shuttle vector	[26]
pBTK	1.5 kb *aph*A3 (Km^R^) allele cloned into *Sma*I site in pBT2ts	[18]
pBTS	1.3 kb *aad*9 (Sp^R^) allele cloned into *Sma*I site in pBT2ts	[18]
pMWO-100	COL C’ CcpA-His_6_ fusion in *Bam*HI/*Kpn*1 of pQE30	This Study
pMWO-101	COL N’ His_6_-Rex fusion in *Nde*I/*Not*1 of pET24b	This Study
pJF102	Δ*rex*::Km^R^ in Shuttle Vector pBT2ts	This Study
pJF103	Δ*ccpA*::Sp^R^ in Shuttle Vector pBT2ts	This Study
pJF115	562 bp full p*_ldh_*_1_ region cloned into pCN52	This Study
pJF116	248 bp p*_ldh_*_1_ region lacking CRE cloned into pCN52	This Study
pJF117	204 bp p*_ldh_*_1_ region lacking CRE and distal Rex site in pCN52	This Study
pJF118	150 bp p*_ldh_*_1_ region lacking CRE and both Rex sites in pCN52	This Study
pJF119	Replaced *Apa*I/*Xho*I Er^R^ region of pCN52 with CAT (Cm^R^)	This Study
pJF120	218 bp p*_rpoD_* region in pJF119	This Study
pJF122	562 bp full p*_ldh_*_1_ region cloned into pJF119	This Study
pAC001	p*_ldh_*_1_CRE^*^ in Shuttle Vector pBT2ts	This Study

Primers	Sequence	
ccpA.1A	5′ CAGGTCGGATCCACAGTTACTATATATGATGTAGC 3′	
ccpA.1B	5′ GATGGTACCTTATTTTGTAGTTCCTCGG 3′	
rex.1A	5′ GGGAATTCCATATGAGTGACCAAGTTAAAATTCC 3′	
rex.1B	5′ ATAGTTTAGCGGCCGCTTCACTGTAATTTTTCATAAAG 3′	
Pldh1.1A	5′ GTGTAAATAATCACTGGCGAAGTACG 3′	
Pldh1.1B	5′ CGCATAACTTAAAAGGTCATGTGTCATCC 3′	
ProcD2.1A	5′ GATTAAGTGTAAAATTATCAATTC 3′	
ProcD2.1B	5′ CTCCCCTTTTCATCATTTATAAC 3′	
NS_band.1A	5′ TGACTTTAGTGAATTTACACCAGG 3′	
NS_band.1B	5′ CGTTTAACGCCAAAAGTTAAATGG 3′	
ccpA-5′.1A	5′ CGACTCTAGAGGATCCTTCGAGTTATTAAAAGAAGCTGGCCG 3′	
ccpA-5′.1B	5′ TACTTGCTGGGGATCCAGTAACTGTCATAATTTCCTCCTTGTAAACG 3′	
ccpA-3′.1A	5′ TACCGAGCTCGAATTCGAATTGAATACCGAGGAACTACAAAATAAATTC 3′	
ccpA-3′.1B	5′ CAGTGCAGCGGAATTCCGCTTTAACTTTAGCAGATCTTATTATGTCAG 3′	
rex-5′.1A	5′ ATGCCTGCAGGTCGACCGTAATTACCAACATAGCGTTTGACATCAC 3′	
rex-5′.1B	5′ ATCCTCTAGAGTCGACCATTCGCTATTTCCTCCTTCGTGTTTG 3′	
rex-3′.1A	5′ TACCGAGCTCGAATTCCAGTGAATAAATGTGATGTTAGCTTTGAATG 3′	
rex-3′.1B	5′ CAGTGCAGCGGAATTCTTATACCTAAACGGTCATTTCTTCCAGTTG 3′	
adhE_RT.1A	5′ AAGTGGCAATTATGATTGATGCTC 3′	
adhE_RT.1B	5′ TCATGTGCTAATTTTGCTAGCACC 3′	
ald1_RT.1A	5′ GTGAAGGACGTGTAGCTTGC 3′	
ald1_RT.1B	5′ TCGTGAGTTACGATCTTAGCG 3′	
ddh_RT.1A	5′ CTTACGGTATTAAACAAATTGCAC 3′	
ddh_RT.1B	5′ TAGGGCGATAGAAACAGAATACTC 3′	
ldh1_RT.1A	5′ AAAACATGCCACACCATATTCTCC 3′	
ldh1_RT.1B	5′ TACTAAATCTAAACGTGTTTCTCC 3′	
pflA_RT.1A	5′ AAAAATGGAAGATGGAACAGACAC 3′	
pflA_RT.1B	5′ TCGATAACTGCATTACTTGTTCCC 3′	
rex_RT.1A	5′ CGTAAAAGAAGATGTTATTGGC 3′	
rex_RT.1B	5′ CTGGTGTAGTTAGAATCACAAC 3′	
rpoD_RT.1A	5′ AACTGAATCCAAGTGATCTTAGTG 3′	
rpoD_RT.1B	5′ TCATCACCTTGTTCAATACGTTTG 3′	
rpoD_gfp.1A	5′ TACCGAGCTCGAATTTAGCATGTGATTTTAAAGAATAATACGAATAATG 3′	
rpoD_gfp.1B	5′ ATTAGTTAACGAATTCTCCCGATTTTAAATATGAACATTCG 3′	
ldh1_fusion1.1A	5′TACCGAGCTCGAATTCTTTAATCTGTATATTTTGATTATTCTACTAAAAATTTC3’	
ldh1_fusion2.1A	5′ TACCGAGCTCGAATTAGGTGTAAAAATTAAAATAAAATGTGAAATAAATCAC 3′	
ldh1_fusion3.1A	5′ TACCGAGCTCGAATTTATTGACCCAGTACTTAATGCATG 3′	
ldh1_fusion4.1A	5′ TACCGAGCTCGAATTCTTAAAAGCGGATGACACATGAC 3′	
ldh1_fusions.1B	5′ ATTAGTTAACGAATTCAAAAACTCCCTTATGATTAATTCACTAAC 3′	
ldh1_RT^−136^.1A	5′ GAAATAAATCACAAACTTAAAAGCGGATG 3′	
ldh1_RT^−176^.1A	5′ CCCAGTACTTAATGCATGTTAC 3′	
ldh1_UTR_RT.1B	5′ GTTTAATGCTGCTTATTGACGATAC 3′	
CRE_mut.FOR	5′ GTACGAAGACTAAAGACATCTAAGACTTGATCGTACCGGAATTAAAAAGGTGTAAAAA 3′	
CRE_mut.REV	5′ TTTTTACACCTTTTTAATTCCGGTACGATCAAGTCTTAGATGTCTTTAGTCTTCGTAC 3′	
hmp_ldh1.5′	5′ CGTTTGTTTGATAATGTCTTTCTCTTGT 3′	
hmp_ldh1.3′	5′ TACAAAAACTCCCTTATGATTAATTCAC 3′	

### Cloning, Mutant Construction and Reporter Fusions

Promoter::GFP fusions were constructed by directionally cloning PCR fragments into the *Eco*RI site of pCN52 (promoterless Gfp fusion vector) using Infusion® technology (Clontech). Fusions with p*_ldh_*_1_ (fusions 1 through 4) as well as the p*_rpoD_* fusion were generated by amplifying fragments from *S. aureus* COL genomic DNA using primers ldh1_fusion1.1A/ldh1_fusions.1B, ldh1_fusion2.1A/ldh1_fusions.1B, ldh1_fusion3.1A/ldh1_fusions.1B, ldh1_fusion4.1A/ldh1_fusions.1B and rpoD_gfp.1A/rpoD_gfp.1B, yielding pJF115, pJF116, pJF117, pJF118 and pJF120, respectively. Promoter fusions for p*_rpoD_* and p*_ldh_*_1_ (full length promoter fragment) were also generated in pJF119 in which the *Apa*I/*Xho*I fragment of pCN52 harboring the *ermC* gene was replaced with a fragment encoding chloramphenicol acetyltransferase (CAT) yielding pJF120 and pJF122, respectively. CRE* was generated by amplifying *S. aureus* COL genomic DNA with primers hmp_ldh1.5′/CRE_mut.1B and CRE_mut.1A/hmp_ldh.3′. The two resulting amplimers were combined using overlapping PCR using primers hmp_ldh.5′/hmp-ldh.3′. The resulting fragment was cloned into pCR BluntII Topo® (Invitrogen) from which the *Eco*RI fragment harboring CRE* was moved into pBT2ts. Allelic replacement was performed as described previously [18]. Δ*rex* and Δ*ccpA* were generated by cloning 5′- and 3′-homology regions for each gene on either side of a Km^R^ (*rex*) or Sp^R^ (*ccpA*) cassette in the pBTK and pBTS yielding pJF102 and pJF103, respectively. Mutants were made using an allelic replacement scheme previously described [18].

### Quantitative Reverse Transcriptase Real Time PCR (Q RT-PCR)

Cells were grown to OD_660_ = 0.5 and either treated with NO· (2 mM DEA-NO, AG Scientific) for 15 minutes or left untreated. Twenty-five ml of culture was added to an equal volume of ice cold ethanol:acetone (1∶1) and incubated at −80°C until further use. All frozen cell suspensions were thawed at room temperature, pelleted by centrifugation and resuspended in 500 µl TE for mechanical disruption using Lysing Matrix B (MP Biomedicals, Solon, OH) in a standard cell disruptor. One-hundred µl of lysates were used for RNA isolation using an RNAEasy® Mini Kit (Qiagen, Valencia, CA) per manufacturer instructions. RNA was spectrophotometrically quantified and 50 ng of total RNA analyzed per reaction using the Sensimix™ SYBR & Fluorescein One-Step kit (Bioline). Reaction conditions were as specified by Bioline and performed on a MyIQ Single color Real-Time PCR Detection System (BioRad). Primers used for analysis are listed in Table 1. All transcript levels were normalized to those of *rpoD*, which exhibited little variation across the growth conditions described here. Transcriptional start site for p*ldh*1 was tested using RT-PCR by amplifying either cDNA or genomic DNA with primers ldh1_RT^−136^.1A/ldh1_UTR_RT.1B and ldh1_RT^−176^.1A/ldh1_UTR_RT.1B (Table 1). Statistical significance was determined using Student’s t-test (2-tailed).

### Electromobility Shift Assays (EMSAs)

CcpA and Rex were purified as N’-terminal and C’-terminal His_6_ tagged fusions, respectively. CcpA was amplified from *S. aureus* COL using primers ccpA.1A/.1B (Table 1) and cloned into the *Bam*HI/*Kpn*I sites of pQE-30 (Qiagen) creating plasmid pMWO-100. Rex was amplified from *S. aureus* COL using primers rex.1A/.1B (Table 1) and cloned into the *Nde*I/*Not*I sites of pET-24b (Novagen) to generate pMWO-101. pMWO-100 was maintained in *E. coli* XL-1 Blue with appropriate selection whereas pMWO-101 was transformed into *E. coli* BL21 and maintained with appropriate selection. Cultures were inoculated into 500 mL LB medium and cultivated at 37°C until OD_600_ = 0.4, at such time cultures were induced with 0.5 mM IPTG shaking at 37°C for 5 h. Cells were then pelleted, resuspended in binding buffer (20 mM Tris, pH 8.0, 500 mM NaCL, 10% glycerol, 5 mM imidazole) then lysed via sonication. Cell debris was pelleted via centrifugation and the culture supernatants applied to an equilibrated Ni-NTA column (Qiagen). The column was then washed and eluted in binding buffer with increasing concentrations of imidazole up to 1 M. Eluates were combined and passed over a Superdex 75 gel filtration column (GE Healthcare) for further purification and buffer exchange against 10 mM Tris-HCl, pH 7.5, 100 mM NaCl. Protein was concentrated using a Microcon centrifugal concentrator (Millipore) and glycerol was added to 10% final concentration prior to flash freezing and storage at −80°C. Final protein concentrations were then determined using a Bradford Assay (BioRad). Protein was incubated at room temperature with 250 fmol of both specific and non-specific probe DNA at varying molar ratios for 15 minutes prior to loading. CcpA::DNA mixtures were loaded onto a pre-run (1 h at 75 V) 8.0% Polyacrylamide gel (1X TBE) and run ice-cooled at 125 V. Rex::DNA mixtures were similarly run through 7.0% polyacrylamide gels. Gels were stained 1∶10,000 with GelRed (Biotum) for 5 min, destained with deionized H_2_O and then imaged via UV-exposure on a GelDoc system (BioRad).

### Ldh1 Enzyme Activities

Cultures of *S. aureus* Δ*ldh*2 strains were grown in 1 L volumes in BHI medium to OD_660_ = 1.0 then aliquoted into 250 ml sealed Sorvall centrifuge tubes and incubated at 37°C for 2 h. Cells were pelleted, disrupted mechanically using Lysing Matrix B (MP Biomedicals, Solon, OH) and then cell debris was removed via centrifugation. Protein concentration for each lysate was determined using a BCA method (Pierce, Rockford, IL). Ldh1 reaction mixtures contained 1 mg total protein from cell-free extracts, 100 mM Tris·Cl pH8.5, and 3 mM NAD^+^. Reactions were initiated by the addition of 13.9 mM L-lactate. Ldh1 activity was defined as the L-lactate-dependent reduction of NAD^+^, which was monitored spectrophotometrically at 340 nm (mM ξ_340_ for NADH = 6.2). Significance was determined using Student’s t-test (2-tailed).

### Metabolite Analyses

Extracellular ammonia and glucose levels in culture supernatants were determined enzymatically using commercially available kits (R-Biopharm). Culture samples were heat inactivated at 55°C for 5 min, cells were pelleted and supernatants were used for analyses. NAD^+^/NADH ratios were also determined using commercially available kits (BioVision). Cells were cultured in PN defined medium with glucose and Casamino Acid carbon sources (0.5% each). Aerated cultures (50 ml in 250 ml Erlenmeyer flasks at 250 rpm) were grown to an OD_660_ = 1.0 then immediately filtered through a 0.45 µm nylon filter (GE Health). Alternatively, NO·-treated cultures were exposed to 2 mM DETA-NO for 20 minutes prior to filtering. Cells were harvested from the nylon filter and resuspended in 600 µl of cold NAD Extraction Buffer (BioVision), flash frozen in a dry ice/EtOH bath, thawed and then mechanically disrupted via lysing matrix B (MP Biomedicals). Cell debris was removed via centrifugation and supernatants were assayed for NAD^+^ and NADH levels as per manufacturer instructions (BioVision). Statistical significance was determined using Student’s t-test (2-tailed).

## Results

### NO·-mediated Induction of Ldh1 Activity Relies on the Presence of Glucose

**Figure 1:**
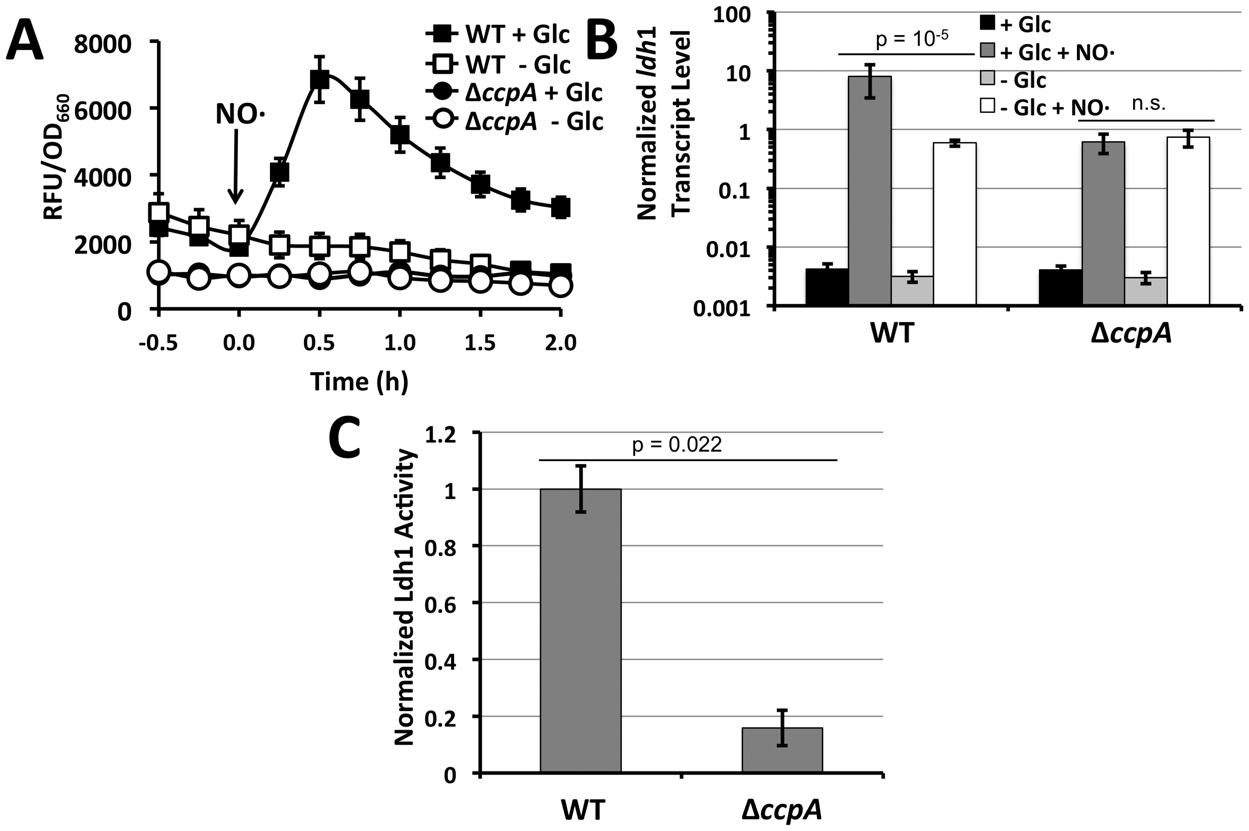
Ldh1 expression in *S. aureus* is dependent on glucose and CcpA. **A.** WT *S. aureus* strain Newman and an isogenic Δ*ccpA* mutant harboring P*_ldh_*_1_::GFP promoter fusions were grown in chemically defined medium with 0.5% casamino acids as primary carbon/energy sources. Glucose (0.5%) was added when indicated. Once cultures reached early exponential phase, NO· was administered (1 mM DETA/NO) and fluorescence and optical density were monitored for two hours. **B.** Quantitative Real-Time Reverse Transcriptase PCR (Q RT-PCR) was used to determine *ldh*1 transcript levels relative to *rpoD* in WT *S. aureus* strain COL and an isogenic isogenic Δ*ccpA* mutant grown in media as described in Figure 1A. NO· was administerd as 2 mM DEA-NO. **C.** Ldh1 enzyme activity from cell extracts of WT and isogenic Δ*ccpA S. aureus* strain COL lacking *ldh*2. Cells were cultured in BHI and stimulated with NO·(2 mM DEA-NO) 15 minutes prior to obtaining lysates.

Exposing *S. aureus* strain COL to NO· in a chemically defined medium with casamino acids as the primary carbon source did not result in detectible induction of a P*_ldh_*_1_::GFP fusion (Figure 1A). However, supplementation of this defined medium with 0.5% glucose restored robust P*_ldh_*_1_::GFP expression upon NO·-exposure. In contrast, the stimulatory effect of glucose was not observed in the isogenic Δ*ccpA* mutant (Figure 1A). Using quantitative real-time PCR to assess transcript levels from chromosomal *ldh*1, a >3-log induction in *ldh*1 transcript levels upon NO·-stimulation in the presence of glucose was observed relative to *rpoD* (Figure 1B). This induction was diminished by more than 13-fold when *S. aureus* COL was exposed to NO· in media devoid of glucose. Again, similar to what was observed with the GFP promoter fusions, the positive effect of glucose on *ldh*1 transcript levels was not apparent in the Δ*ccpA* mutant (Figure 1B). It should be noted, that in aerobic environments in the absence of NO·, *ldh*1 transcript is barely detectible resulting in large apparent induction ratios upon NO·-exposure. Thus, in the absence of glucose and/or CcpA, *ldh*1 transcript levels were still effectively induced ∼200-fold by NO· (Figure 1B). However, in comparison to WT cells grown in the presence of glucose, the >1-log reduction in overall *ldh*1 transcript in the absence of glucose and/or CcpA correlates with the absence of detectible GFP signal on a multi-copy promoter fusion (Figure 1A). Likewise, measuring Ldh1 enzyme activity revealed that without CcpA, Ldh1 activity is barely above background implying that without glucose stimulation, *S. aureus* effectively lacks Ldh1 activity despite detectible increases in transcript (Figure 1C).

### Glucose-dependent Stimulation of *ldh*1 Requires both CcpA and Rex

**Figure 2:**
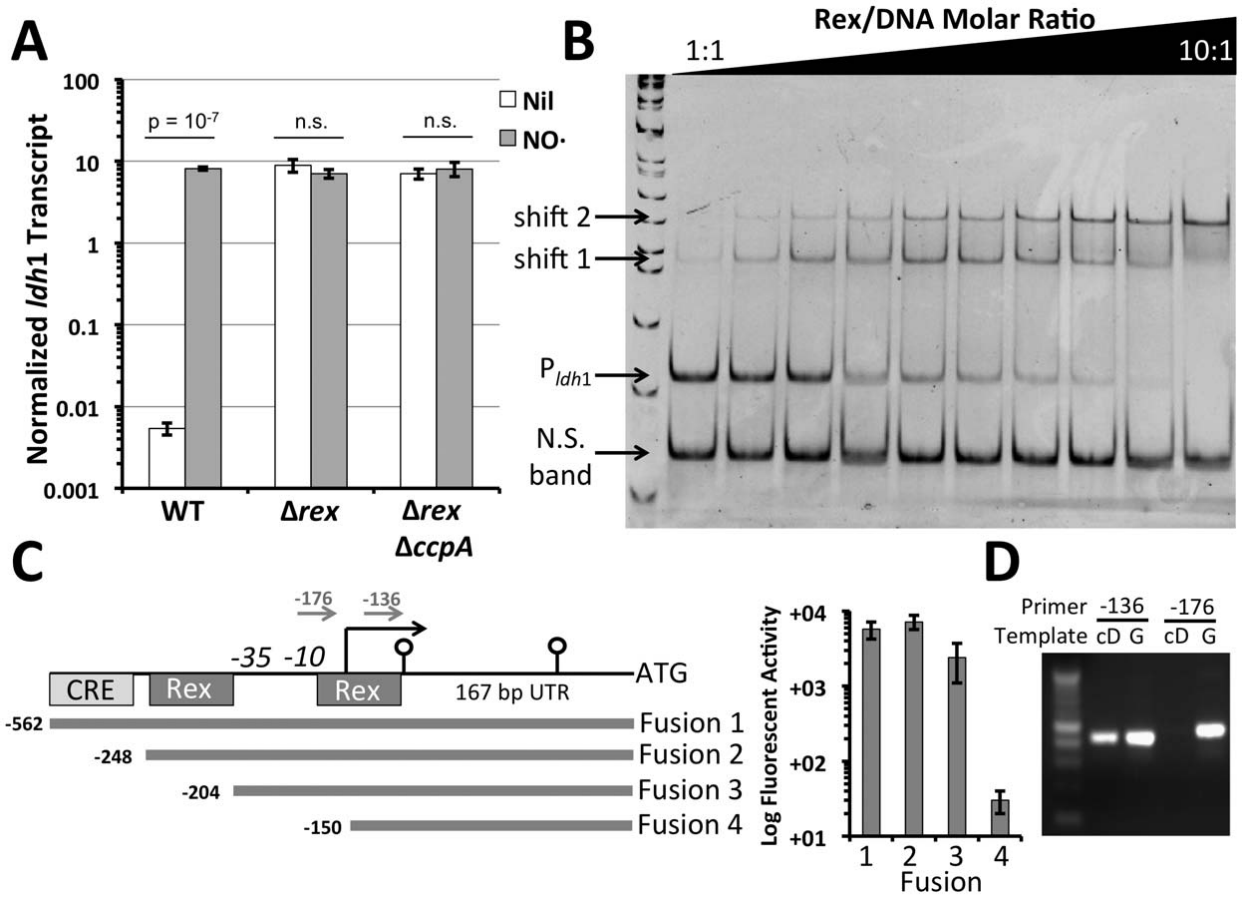
Both Rex and CcpA are required for glucose-mediated induction of *ldh*1. **A**. Q RT-PCR of *ldh1* transcript from *S. aureus* strain COL normalized to *rpoD* in cells exposed/unexposed to NO· administered as 2 mM DEA-NO 15 minutes prior to RNA isolation. **B**. ElectroMobility Shift Assay (EMSA) of P_*ldh1*_ using purified His-Rex at increasing molar ratios of Rex:DNA (250 fmol promoter DNA in all wells). Internal fragment of *hmp* was used as a non-specific probe (N.S. band). As predicted by the presence of two Rex sites in P_*ldh1*_, two independent shifted bands appear with increasing Rex::DNA molar ratios. **C**. Schematic representation of P_*ldh1*_ and fragments used for GFP:fusions and their relative activity following stimulation with NO· (2 mM DETA-NO). The activity of Fusion 4 was indistinguishable form that of a promoterless control. **D**. Reverse transcriptase PCR using two different forward primers depicted in Figure 1C to amplify products using both cDNA (cD) and genomic (G) DNA as templates.

In response to NO·, the high level induction of *ldh*1 transcript in the presence of glucose was identical to the basal *ldh*1 transcript level in the Δ*rex* mutant (Figure 2A). Furthermore, exposure to NO· did not affect *ldh*1 transcript levels in the Δ*rex* background. Additionally, inactivation of *ccpA* in the Δ*rex* background did not attenuate the constitutive expression of *ldh*1 (Figure 1B). These data imply that CcpA does not activate *ldh*1 independently of Rex-mediated repression. Thus, glucose-dependent induction of *ldh*1 relies on the presence of both Rex and CcpA, but CcpA must affect the ability of Rex to repress *ldh*1 transcription.

Two putative Rex binding sites (TGTGAWWWWWWTCACA) can be identified −160 bp and −218 bp upstream of the *ldh*1 start-codon. Purified Rex bound specifically to both sites *in vitro* consistent with the role of Rex in *ldh*1 repression (Figure 2B). Interestingly, a similar consensus sequence has been proposed for the iron-sulfur cluster containing oxygen sensor ArcR [19]. However, deletion of ArcR had no affect on *ldh*1 expression under anaerobiosis (data not shown, [10]). Furthermore, the Δ*arcR* mutant exhibited no alterations in the expression of other Rex-regulated genes including *pflA*, *adhE* or *ddh* implying that the two regulators do not share identical binding sites and that Rex, not ArcR, controls *ldh*1 expression.

The fact that the putative Rex binding sites were between 160 and 218 bp upstream of the *ldh*1 coding sequence prompted us to map the transcriptional start site of *ldh*1 using 5′-RACE. However, given the apparent secondary structure predicted to occur within the 5′UTR of the *ldh*1 transcript, 5′RACE was unsuccessful at identifying the start of *ldh*1 transcription (Figure 2C). By using a combination of *in silico* prediction models combined with truncated GFP-promoter fusions and Reverse-Transcriptase PCR we established that the start of *ldh*1 transcription was likely within the proximal Rex binding site (Figure 2C). That is, a reverse oriented primer within *ldh*1 was able to amplify a product (later verified by sequencing) with a forward-oriented primer positioned at −136 from *ldh*1 using both genomic and cDNA templates (Figure 2D). In contrast, using a forward-oriented primer positioned at −176 from the *ldh*1 ATG was unable to amplify a product specifically from cDNA. Furthermore, P*_ldh_*_1_::GFP promoter fusions were highly active until the cloned fragment was truncated past −150 relative to *ldh*1 ATG (Figure 2C). Thus, the 26 bp fragment from −150 to −176 likely contains the *ldh*1 transcriptional start site. A putative −10 and −35 sequence could be identified upstream of this region putting the *ldh*1 transcriptional start site at the guanine residue −167 bp upstream of the *ldh*1 start codon. Consistent with Rex-mediated *ldh*1 repression, this start site lies within the TGTGA inverted repeat of the *ldh*1-proximal Rex binding site.

### The CcpA-dependent Glucose Effect on *ldh*1 Expression does not Involve Altered Rex Activity

**Figure 3:**
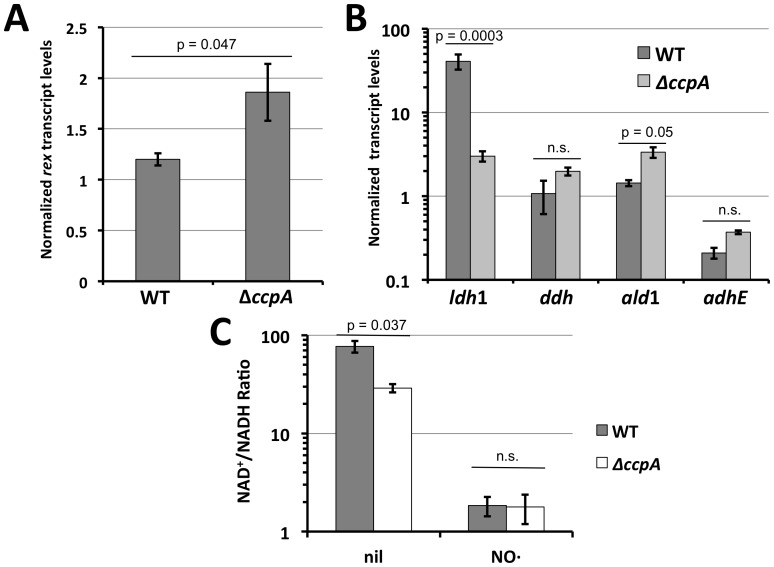
Alteration of Rex levels and/or activity cannot explain the Rex-dependency of ldh1 glucose-induction. **A.** Q RT-PCR analyses of *rex* transcript levels normalized to those of *rpoD* in WT versus Δ*ccpA S. aureus* strain COL following NO· stimulation. **B.** Q RT-PCR analyses of other Rex-regulated genes normalized to *rpoD* upon NO·-stimulation. Only *ldh*1 exhibits CcpA-dependent activation. **C.** Redox status depicted as NAD^+^/NADH ratios of WT versus Δ*ccpA S. aureus* COL prior to and after stimulation with NO·.

The fact that the presence of glucose stimulates *ldh*1 transcription in a manner dependent on both Rex and CcpA suggests that CcpA might affect Rex activity thereby modulating *ldh*1 expression. Indeed, *rex* transcript levels were a modest 50% increased in the Δ*ccpA* background (Figure 3A). Higher Rex levels could be predictive of more *ldh*1-repression as seen in Figure 1B. However, this modest increase in *rex* transcript did not result in reduced expression of other Rex-regulated genes including *ddh*, *ald*1 and *adhE*. Alternatively, the Δ*ccpA* mutant may exhibit less redox-imbalance under NO·-stress thereby increasing the repressive effects of Rex. That is, Rex activity should be more pronounced in conditions of redox balance with relatively low NADH levels (*e.g.* high NAD^+^/NADH ratios). However, upon exposure to NO· the Δ*ccpA* mutant exhibited similar redox imbalance compared to WT (same NAD^+^/NADH ratios) (Figure 3C). In unexposed cells, Δ*ccpA* mutants actually exhibited enhanced redox imbalance (lower NAD^+^/NADH ratios). This should act to further relieve Rex-mediated repression, however Ldh1 activity is diminished in a Rex-dependent fashion in this strain (Figures 1C and 2A). Thus, CcpA must affect the ability of Rex to repress Ldh1 in a manner that does not alter Rex protein levels or activity. One hypothesis is that CcpA binding to the CRE site upstream of the distal Rex binding site reduces the ability of Rex to bind DNA and therefore repress *ldh*1 transcription.

### CcpA does not Interact Directly with the *ldh*1 Promoter but rather Acts Indirectly by Promoting Glucose Utilization

**Figure 4:**
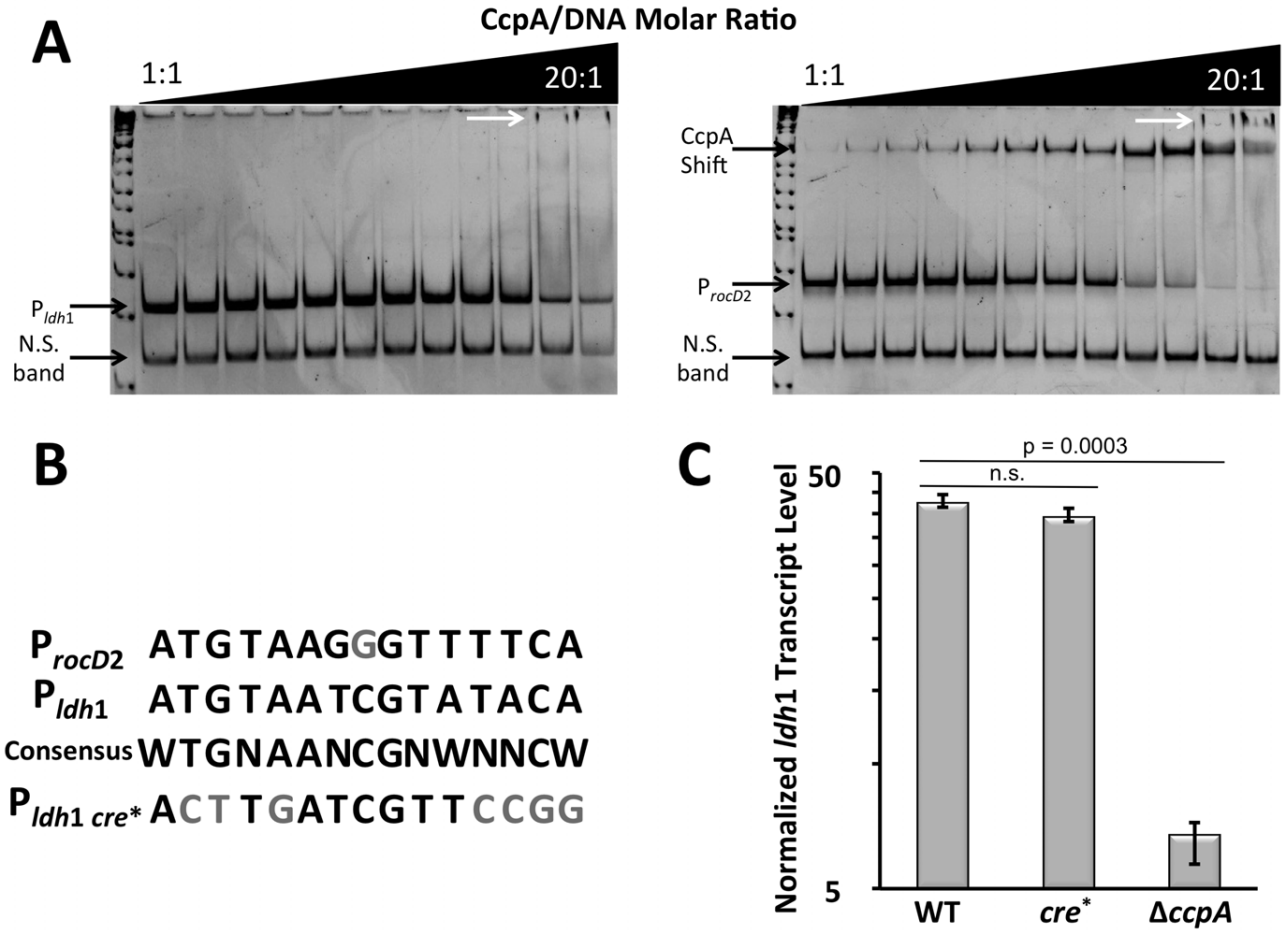
CcpA affect at P*_ldh1_* is indirect. **A.** EMSA with His-tagged CcpA using P*_ldh_*_1_ (LEFT) or P*_rocD_*_2_ (RIGHT) as probes and an internal *hmp* fragment as a non-specific probe (N.S. band). Only at highest ratios of CcpA::DNA did non-specific shifted bands become evident using P*_ldh_*_1_ as a probe (white arrows). 250 fmol of DNA probes were used in all wells. **B.** Alignment of CRE from P*_ldh_*_1_, P*_rocD_*_2_, the *B. subtilis* consensus sequence and the mutated CRE^*^. **C.** Q RT-PCR analyses of *ldh*1 transcript levels normalized to those of *rpoD* in WT, Δ*ccpA* and CRE^*^ derivatives of *S. aureus* strain COL following 15 min. NO· exposure (2 mM DEA-NO).

We sought to show that CcpA binding to the CRE site near P*_ldh_*_1_ could occlude Rex from the promoter and relieve *ldh*1 repression. However, purified CcpA showed no affinity for P*_ldh_*_1_ over that of non-specific DNA probe (Figure 4A). In contrast, purified CcpA was able to shift the *rocD*2 promoter, a DNA fragment know to be directly bound by CcpA [16]. The lack of CcpA binding to P*_ldh_*_1_ was surprising given that the putative CRE site upstream of *ldh*1 was completely consistent with the proposed Rex-consensus sequence from *B. subtilis* (Figure 4B). CcpA normally binds with higher affinity to CRE sites when complexed with P∼Ser_46_-HPr, so an attempt to show *in vivo* CcpA interactions with the CRE of P*_ldh_*_1_ was undertaken. Despite altering seven key base pairs of the CRE consensus (CRE^*^) in the genomic P*_ldh_*_1_, there was no measurable effect of CRE^*^ on *ldh*1 transcription (Figure 4C). Similarly, the chromosomal CRE^*^ mutation had no affect on Ldh1 enzyme activity in *S. aureus* COL (data not shown). Thus, CcpA does not directly bind to the predicted CRE site of P*_ldh_*_1_ *in vitro* and the CRE site upstream of *ldh*1 exerts no measurable effects on Ldh1 expression *in vivo.*

**Figure 5:**
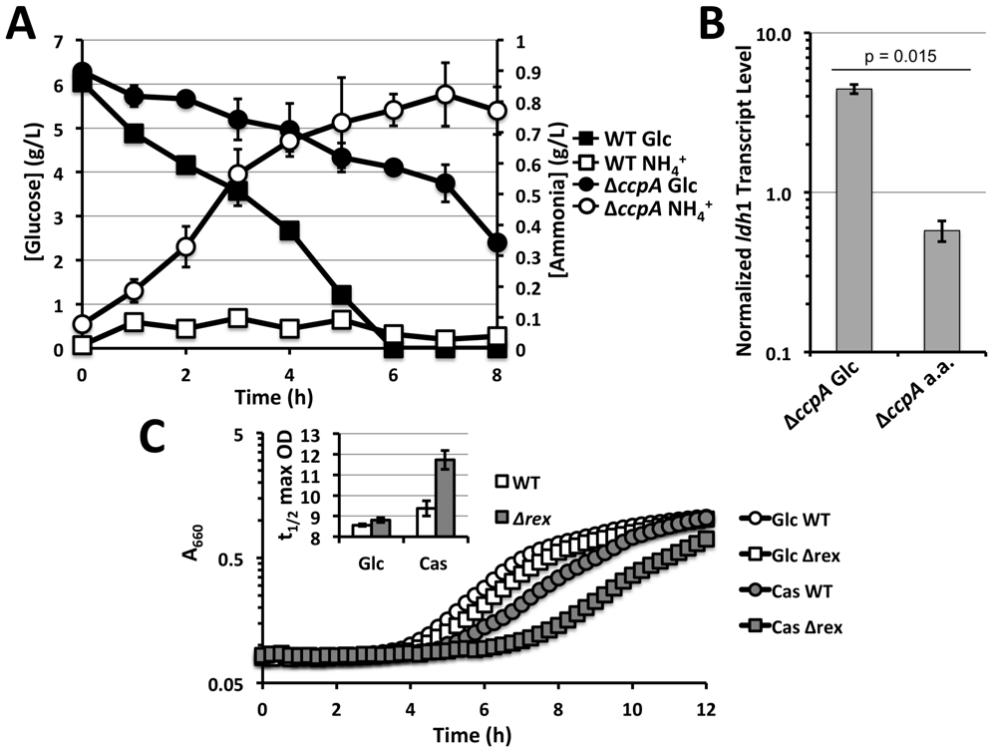
Performing glycolysis stimulates *ldh*1 expression. **A.** Metabolite analyses of WT *S. aureus* COL and an isogenic Δ*ccpA* mutant grown in defined medium devoid of ammonia with both glucose (0.5%) and casamino acids (0.5%) as carbon sources. Glucose utilization is delayed and ammonia production is enhanced in the Δ*ccpA* mutant implying altered carbon source preference. **B.** Q RT-PCR analyses of *ldh*1 transcript levels normalized to those of *rpoD* in Δ*ccpA S. aureus* COL following 15 min NO·-exposure (2 mM DEA-NO) cultivated in defined medium with indicated carbon source. **C.** Representative growth curve demonstrating the defect of a Δ*rex* mutant compared to isogenic WT *S. aureus* COL when utilizing gluconeogenic carbon sources. Inset: average time to ½ maximum optical density as a metric for length of lag phase from four independent curves.

Together, these data suggest that the effect of CcpA on *ldh*1 expression is indirect. We therefore wanted to determine whether the glucose-mediated activation of Ldh1 was dependent on CcpA at all (Figure 1). Since Δ*ccpA* mutants grow poorly in chemically defined medium with glucose alone as the primary carbon source, throughout this study WT and Δ*ccpA* strains were grown in media with casamino acids as carbon sources supplemented with or without 0.5% glucose (Figure S2). In media supplemented with both casamino acids and glucose, WT cells preferentially consume glucose with little ammonia production indicating limited consumption of amino acids (Figure 5A). In contrast, Δ*ccpA* mutants actively produced ammonia and consumed less glucose compared to WT. Thus, a Δ*ccpA* mutant primarily consumes amino acids in this defined medium whereas WT preferentially performs glycolysis. When “forcing” the Δ*ccpA* mutant to utilize glucose by growing in defined medium with no casamino acids, *ldh*1 was expressed eight-fold higher compared to growth on casamino acids alone (Figure 5B). This increased expression was evident despite poor growth rates of Δ*ccpA* on glucose (Figure S2). These results indicate that utilization of glucose promotes Ldh1 expression independently of CcpA. Furthermore, the deficiency in glucose utilization observed in the Δ*ccpA* mutant can explain the apparent dependency of Ldh1 expression on CcpA.

### The Glucose-requirement for Maximal Ldh1 Activation Prevents Carbon Loss during Gluconeogenesis

Lactate production via Ldh1 provides redox balance by re-oxidizing NADH produced during glycolytic conversion of carbohydrates to pyruvate. However, when carbohydrates are scarce, *S. aureus* can utilize amino acids, lactate, pyruvate and other gluconeogenic substrates for carbon and/or energy. Under these conditions, high level Ldh activity would be detrimental since pyruvate pools would need to be converted to phosphoenopyruvate (PEP) via oxaloacetate for gluconeogenesis. High Ldh activity would effectively compete for available pyruvate, diminishing flux through gluconeogenesis. The Δ*rex* mutant expresses high Ldh1 activity irrespective of the presence/absence of glucose (Figure 2A). Likewise, Δ*rex* mutants have difficulty growing in media with pyruvate or casamino acids as a sole carbon/energy sources (Figure 5C). That is, Δ*rex* mutants exhibit an extended lag phase (∼2.5 h) only on gluconeogenic carbon sources such as amino acids and pyruvate. Thus, the conserved dependence on glucose catabolism in bacteria for maximal Ldh activity, either transcriptionally as in *S. aureus* or posttranscriptionally as in many other bacteria, represents a fundamental control mechanism ensuring efficient carbon utilization in the absence of abundant carbohydrates.

## Discussion

The ability to resist NO·-mediated toxicity separates *S. aureus* from most other bacteria including coagulase-negative staphylococcal species [6]. A major difference in the *S. aureus* NO·-response compared to other bacteria is the metabolic adaptations mounted by this pathogen against nitrosative stress [4], [5]. Glucose has been shown to be essential for *S. aureus* NO·-resistance [18]. There are several possible explanations as to why glycolysis is the primary central metabolic pathway used by *S. aureus* under NO·-stress. For instance, NO· may target key gluconeogenic enzymes limiting use of non-glycolytic carbon/energy sources. Alternatively, even with a full repertoire of active non-glycolytic pathways, gluconeogenic metabolism may simply be incompatible with the effects of NO· (*e.g.* elicits excessive redox imbalance). On the other hand, glycolysis may be required for the induction of key metabolic enzymes in *S. aureus* during growth in the presence of NO·. Here we demonstrate that, at least in the case of *ldh*1, glucose stimulates its expression and is required for full Ldh1 activity. Given that Ldh1 is the primary redox-balancing enzyme for *S. aureus* during periods of diminished respiratory activity, reduced *ldh*1 transcription in the absence of glucose may partially account for inability of *S. aureus* to thrive under NO·-stress on gluconeogenic carbon/energy sources.

*S. aureus* acquired *ldh*1 after emerging evolutionarily from other staphylococci given that the allele can only be found in *S. aureus* genomes where it seems to be universally present. Transcript levels of *ldh*1 are kept virtually undetectable in respiring cells by the direct binding of the Rex repressor to two Rex-sites within P*_ldh_*_1_ (Figure 2). Thus, Ldh1 activity is only detected in redox-stressed cells (increased NADH levels). Here we show, as others have suggested [12], that maximal Ldh1 activity also relies on the presence of glucose. It is interesting that of all the tested Rex-regulated genes, only *ldh*1 appears to be under glucose/CcpA control (Figure 1B). This creates a scenario whereby in the presence of glucose, *S. aureus* preferentially balances redox by the production of L-lactate. This is not the case in other staphylococcal species, which produce commensurate levels of D- and L-lactate anaerobically [6]. The “enantiomer preference” of *S. aureus* may stem from the fact that L-lactate alone can be utilized by lactate-quinone oxidoreductase (Lqo) [18]. Thus, L-lactate may represent more of a metabolic intermediate than D-lactate, which is primarily a metabolic endproduct. However, the true selective advantage of L-lactate production over D-lactate in *S. aureus* is still unknown.

The fact that glucose-stimulation of Ldh1 expression required the presence of Rex implies that glucose somehow modulates that ability of Rex to repress *ldh*1 transcription. While the Δ*ccpA* mutant produced marginally more *rex* transcript (∼50% increase over WT), the impaired redox balance of the Δ*ccpA* mutant (high NADH levels) would predict that the excess Rex would be less active in these cells (Figure 3). However, Ldh1 activity is barely detectible in non-glucose grown cells or Δ*ccpA* mutants grown even in the presence of glucose. Furthermore, given that the entire Rex regulon did not exhibit glucose-dependent induction implies that the glucose affect on Rex is specific for p*_ldh_*_1_. This does not exclude the possibility that the Rex has higher affinity for sites at the promoters for *ddh*, *adhE*, and *ald*1 and therefore the glucose effect is masked. However, the higher NADH levels in cells cultured on amino acid carbon sources predict that all Rex regulated genes should be derepressed on amino acids. Indeed, *ald*1 did exhibit a significant >2-fold induction on amino acid media compared to glucose consistent with NADH-mediated Rex inactivation (Figure 3B). The fact that *ldh*1 expression behaves in an opposing trend (lower expression on amino acids leading to higher NADH-levels) implies a separate form of regulation. The requirement for Rex to observe glucose-stimulated induction also eliminates the role for a “traditional” activator driving *ldh*1 transcription since that would predict a drop in Ldh1 expression in the Δ*rex* mutant grown in the absence of glucose. Rather, the data suggest that a glucose-responsive regulator is acting as an anti-repressor limiting the ability of Rex to block *ldh*1 transcription (Figure S1). A CRE consensus site ∼30 bp upstream of the promoter distal Rex site seemed to be in a prime position to allow CcpA to serve as an effective anti-repressor against Rex. However, despite the complete conservation of the P*_ldh_*_1_ CRE with the published consensus from *B. subtilis* [20], CcpA has no affinity for P*_ldh_*_1_ either *in vitro* or *in vivo* (Figure 4). This may result from the presence of a T-A basepair at position 7 of the p*_ldh_*_1_ CRE (Figure 4). This position is never occupied by a T in high-affinity CcpA binding sites and only is present in only 3% of low-affinity sites in *B. subtilis* [20]. The p*_ldh_*_1_ CRE is completely conserved among all available sequences (data not shown). Alternatively, the CRE consensus in *S. aureus* may be significantly divergent from that of *Bacillus* spp. More investigation into the sequences of the *S. aureus* CRE required for CcpA binding will explain these curious results.

The fact that the Δ*ccpA* mutant exhibits altered carbon source utilization preferentially oxidizing amino acids over glucose (Figure 5), and the indirect reduction of *ldh*1 transcription in the Δ*ccpA* background suggest that P*_ldh_*_1_ actually responds to the performance of glycolysis (Figure S1). This glycolysis-stimulated Ldh1 expression theory is further supported by the enhanced transcription of *ldh*1 in the Δ*ccpA* mutant grown in glucose relative to amino acids despite the poor growth of Δ*ccpA* on glucose alone (Figures 5 and S2). Carbohydrate utilization would result in reduced pH whereas peptide catabolism would raise local pH given the excessive ammonia production. Perhaps P*_ldh_*_1_ responds to a drop in intracellular pH thereby being indirectly affected by glycolysis. Alternatively, organic acid production by glycolytic fermentation may trigger *ldh*1 transcription. CidR is a regulator known to respond to the presence of very short-chain organic acids (*e.g.* acetate and lactate), however the Δ*cidR* mutant exhibited no reported alterations in *ldh*1 transcription [21]. Thus the mechanism behind enhanced Ldh1 expression in the presence of glucose is still unknown. However, whatever the mechanism, glucose stimulated *ldh*1 transcription in *S. aureus* will mimic species that express fructose bisphosphate activated Ldh enzymes in that maximal activity will only be present in cells that are actively performing glycolysis. This level of regulation is necessary to conserve carbon during growth in the absence of glucose (Figure 5). Thus it would seem evolutionarily advantageous to limit high Ldh activity when grow under low carbohydrate conditions. Many bacteria have evolved allosteric control of Ldh to achieve this regulation [13]. *S. aureus* appears to have evolved to transcriptionally modulate Ldh activity to achieve the same level of control.

## Supporting Information

Figure S1 **Model of** ***S. aureus*** **glucose-dependent** ***ldh*** **1 regulation.** NO· blocks respiration leading to a buildup of NADH (low NAD^+^/NADH ratios), which diminishes Rex DNA binding activity leading to derepression of *ldh*1. The presence of glucose also diminishes the repressive activity of Rex by an unidentified mechanism. CcpA acts to direct *S. aureus* to preferentially utilize glycolytic carbon sources therefor maximizing the glycolytic effect on Rex-repression. Utilization of glycolytic carbon sources leads to increased steady-state levels of fructose 1,6,-bisphosphate (FBP), which signals the phosphorylation of HPr on a conserved Ser residue. HPr-PO_4_ acts as a co-activator with CcpA.
(TIF)

Figure S2 **Growth defect of Δ** ***ccpA S. aureus*** **COL when “forced” to use glucose as a primary carbon/energy source.** Bacteria were cultivated in chemically defined medium with either 0.5% glucose (Glc), 0.5% casamino acid (a.a.) or the combination (both at 0.5%, Glc/a.a.) as primary carbon/energy sources.
(TIF)
