# Supplementary figures and images for: CcpA-Independent Glucose Regulation of Lactate Dehydrogenase 1 in Staphylococcus aureus
Source: PLoS One. 2013 Jan 14;8(1):e54293. doi: 10.1371/journal.pone.0054293 (PMC3544828; doi:10.1371/journal.pone.0054293)

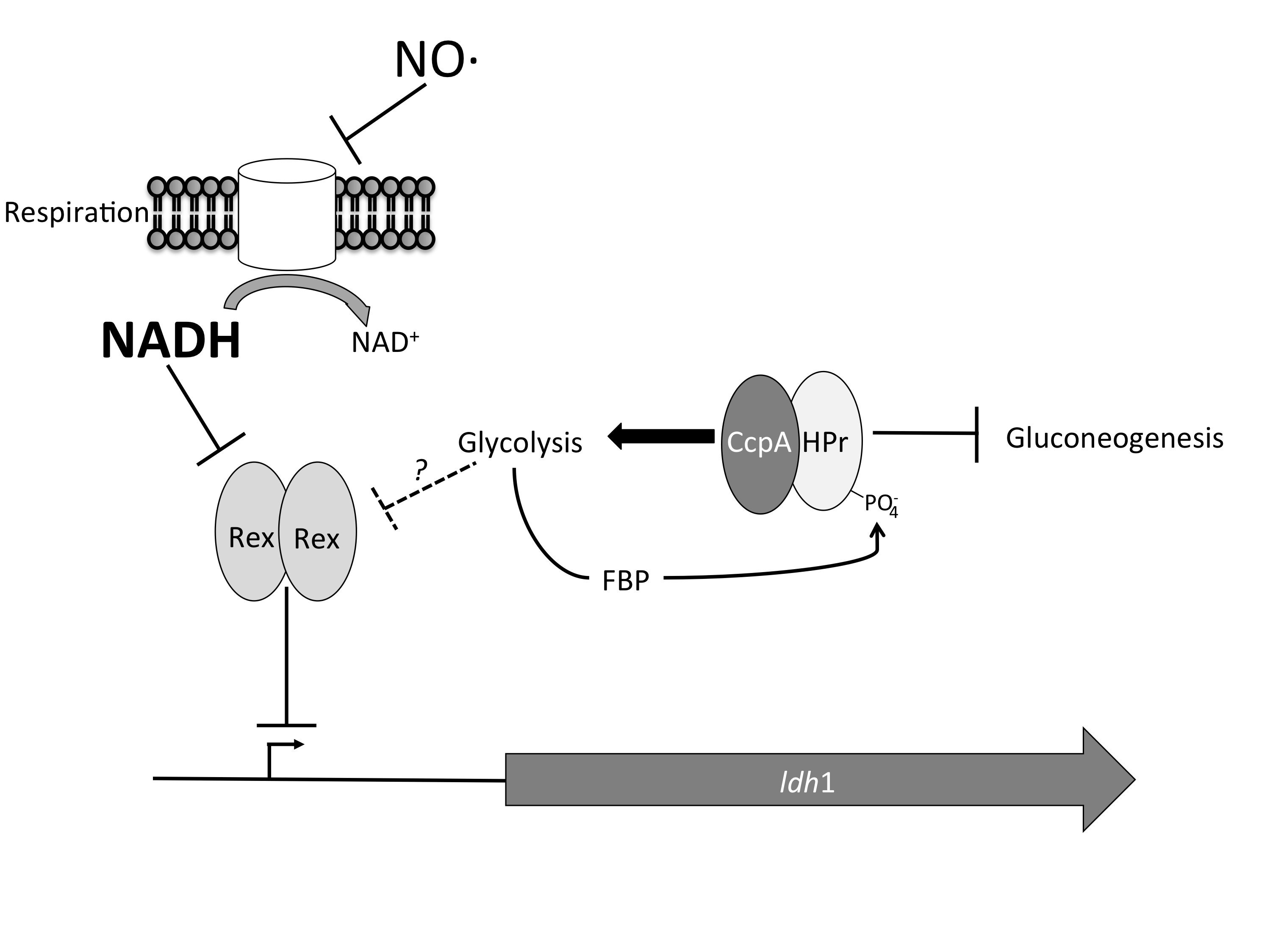

Supplement: Figure S1 — Model of S. aureus glucose-dependent ldh 1 regulation. NO· blocks respiration leading to a buildup of NADH (low NAD+/NADH ratios), which diminishes Rex DNA binding activity leading to derepression of ldh1. The presence of glucose also diminishes the repressive activity of Rex by an unidentified mechanism. CcpA acts to direct S. aureus to preferentially utilize glycolytic carbon sources therefor maximizing the glycolytic effect on Rex-repression. Utilization of glycolytic carbon sources leads to increased steady-state levels of fructose 1,6,-bisphosphate (FBP), which signals the phosphorylation of HPr on a conserved Ser residue. HPr-PO4 acts as a co-activator with CcpA. (TIF) [file pone.0054293.s001.tif]

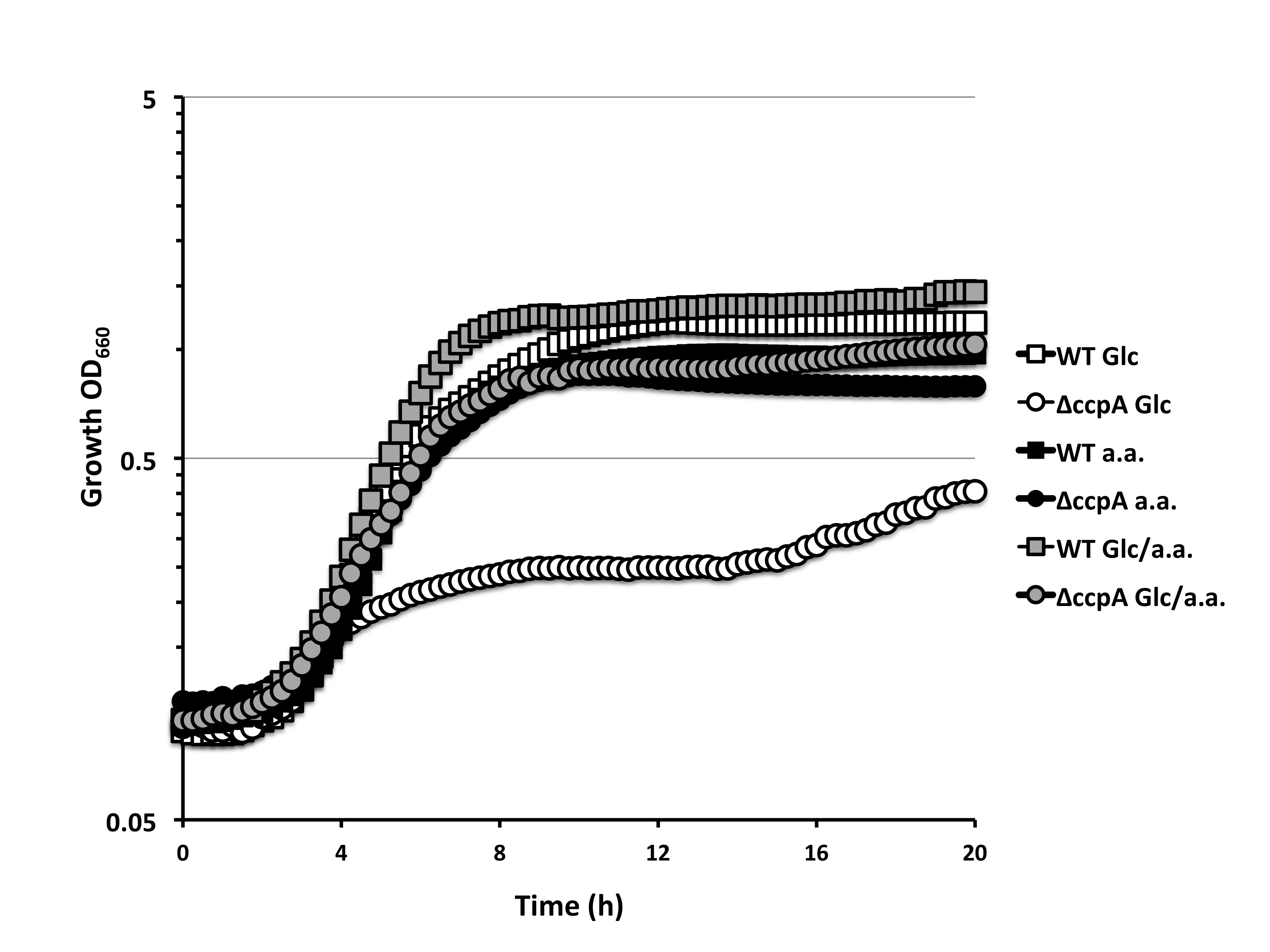

Supplement: Figure S2 — Growth defect of Δ ccpA S. aureus COL when “forced” to use glucose as a primary carbon/energy source. Bacteria were cultivated in chemically defined medium with either 0.5% glucose (Glc), 0.5% casamino acid (a.a.) or the combination (both at 0.5%, Glc/a.a.) as primary carbon/energy sources. (TIF) [file pone.0054293.s002.tif]
